# Efficacy of repetitive transcranial magnetic stimulation (rTMS) adjunctive therapy for major depressive disorder (MDD) after two antidepressant treatment failures: meta-analysis of randomized sham-controlled trials

**DOI:** 10.1186/s12888-023-05033-y

**Published:** 2023-07-27

**Authors:** Róbert György Vida, Eszter Sághy, Richárd Bella, Sándor Kovács, Dalma Erdősi, Judit Józwiak-Hagymásy, Antal Zemplényi, Tamás Tényi, Péter Osváth, Viktor Voros

**Affiliations:** 1 Department of Pharmaceutics, Faculty of Pharmacy, University of Pécs, Pécs, Hungary; 2 Center for Health Technology Assessment and Pharmacoeconomic Research, Faculty of Pharmacy, University of Pécs, Pécs, Hungary; 3 Department of Psychiatry and Psychotherapy, Clinical Center, Medical School, University of Pécs, Pécs, Hungary

**Keywords:** Repetitive transcranial magnetic stimulation (rTMS), Major depressive disorder (MDD), Treatment-resistant depression (TRD), Meta-analysis, Efficacy, Methodology

## Abstract

**Background:**

Several meta-analyses demonstrated the efficacy of unilateral High-Frequency Left-sided (HFL) repetitive Transcranial Magnetic Stimulation (rTMS) for individuals with Major Depressive Disorder (MDD); however, results are contradictory due to heterogeneity of the included studies.

**Methods:**

A systematic literature review (SLR) of English language articles published since 2000 was performed in March 2022 on PubMed and Scopus databases. Empirical evidence on the relative efficacy of rTMS treatment compared with standard pharmacotherapy in Treatment-Resistant Depression (TRD) were extracted. Random effects models were used to assess the effects of rTMS on response and remission rates.

**Results:**

19 randomized double-blinded sham-controlled studies were included for quantitative analysis for response (n = 854 patients) and 9 studies for remission (n = 551 patients). The risk ratio (RR) for response and remission are 2.25 and 2.78, respectively for patients after two treatment failures using rTMS as add-on treatment compared to standard pharmacotherapy. Cochrane’s Q test showed no significant heterogeneity. No publication bias was detected.

**Conclusions:**

rTMS is significantly more effective than sham rTMS in TRD in response and remission outcomes and may be beneficial as an adjunctive treatment in patients with MDD after two treatment failures. This finding is consistent with previous meta-analyses; however, the effect size was smaller than in the formerly published literature.

**Supplementary Information:**

The online version contains supplementary material available at 10.1186/s12888-023-05033-y.

## Background

Depression is a global illness affecting 3.8-5.0% of the adult population and accounting for 280 million cases yearly worldwide [1]. Major Depressive Disorder (MDD) is the fourth leading cause of global disease burden, especially Treatment-Resistant Depression (TRD) have significant socio-economic consequences detectable in reduced work productivity and greater health-care resource use (HCRU) [2].

There are several effective psychopharmacological and psychotherapeutic approaches to treat MDD. However, approximately 50–60% of patients with MDD do not have adequate response to treatment or fail to achieve remission [3, 4]. Within this heterogeneous population, the treatment of patients who meet the well-defined criteria of TRD is a major challenge. The European Medicines Agency (EMA) defined TRD as an unsatisfactory response to two adequate trials of two different classes of antidepressants at the adequate dosage for a sufficient duration [5, 6]. The proportion of TRD among MDD is between 4 and 20% based on prescription registers and literature data [7, 8].

Beyond currently available pharmacological and psychotherapeutic modalities, several neuro-modulation methods, including repetitive Transcranial Magnetic Stimulation (rTMS) have been applied for the treatment of TRD. rTMS utilizes an electromagnet to generate local magnetic field pulses to modulate the activity of local neural circuits and brain function [9]. The advantage of rTMS over other therapies is - in addition to its efficacy in depression - that it may alleviate some elements of cognitive impairment in depression and is more acceptable to patients, considering its adverse-effect profile [10–12].

Numerous studies and meta-analyses have demonstrated the efficacy and cost-effectiveness of rTMS in the treatment of TRD [9, 13] but the validity and the generalizability of these results are limited by several methodological issues. These include the heterogeneous patient population, the limited sample size, the different definitions for TRD, the lack of precise definition of response and remission, the differences in the methods used to evaluate improvement, and the different rTMS treatment protocols and regimens (monotherapy or adjunctive).

To address these methodological issues, a meta-analysis was conducted to collect and analyze all available evidence regarding the application of adjunctive rTMS treatment in the management of TRD, and how different methodological issues may impact the efficacy of add-on rTMS treatment.

## Methods

### Search strategy and study selection

We conducted a systematic literature search in PubMed and Scopus databases for articles published between January, 2000 and March, 2022 with terms and subject headings: repetitive transcranial magnetic stimulation or rTMS, major depressive disorder or treatment resistant depression, sham or sham control, based on the following search query: TITLE-ABS-KEY (rtms OR “repetitive transcranial magnetic stimulation”) AND TITLE-ABS-KEY (“major depressive disorder” OR “treatment resistant depression”) AND TITLE-ABS-KEY (sham OR “sham control”).

We identified Randomized Clinical Trials (RCTs) that were published in English and investigated the relative efficacy of rTMS technology compared with standard pharmacotherapy in adult human population with unipolar TRD. Studies with high-frequency and unilateral application were included for further investigation. We included studies with the number of sessions ranging between 10 and 30 and the number of pulses between 6000 and 120,000. The motor threshold (MT) ranged from 80 to 120%.

Further eligibility criteria included (i) the study was randomized, sham-controlled; (ii) there was information regarding the number of therapeutic failures (and there were at least 2 failed antidepressant treatment); (iii) the severity of depression was assessed with Hamilton Depression Rating Scale (HAM-D/HDRS) [14] or Montgomery-Asberg Depression Rating Scale (MADRS) [15]; (iv) there was information whether the rTMS was used in monotherapy or as adjunctive treatment; (v) the localization of the stimulation was the left dorso-lateral pre-frontal cortex (DLPFC); and (vi) the range of stimulation frequency was reported in the study (5, 10, 20 Hz).

We excluded studies that evaluated different application or indication of the rTMS or if the TRD was accompanied with specific conditions or events (e.g., post-stroke depression, depression related to substance use disorder, etc.). Also, we did not include studies with no reported outcome data.

### Data abstraction, validity assessment

The following information and parameters were extracted by two authors independently: publication year, study design (sham-controlled, monotherapy or augmentation), stimulation frequency, motor threshold, number of therapeutic failures, number of sessions, number of pulses, applied depression scale, age, sex, number of subjects, remission and response rates in active rTMS arm and sham rTMS arm. Any disagreement or uncertainty regarding inclusion of the study and data abstraction was resolved by consensus and feedback from a third team member.

Response rate was defined as the number of subjects that had a minimum 50% or more reduction in post-treatment scores on the Hamilton Depression Rating Scale (HAM-D/HDRS) or on the Montgomery–Asberg Depression Rating Scale (MADRS) [14, 15].

Remission rate was defined as the number of subjects assessed as being in remission based on psychometric testing results. These varied based on the assessment tools used, however defined in terms of a cut-off point, generally as MADRS < 10 and/or HAM-D/HDRS ≤ 7 points. In contrast to MADRS, where the cut-off score for remission is less then 10 points, there was a heterogeneity between the different studies in terms of defining remission by HAM-D/HDRS. Remission was assessed by 9 studies, in which the cut-off value for remission was defined as ≤ 7 in 5 studies [16–20], ≤ 8 in 2 studies [21, 22] and ≤ 10 in one study [23] using HAM-D/HDRS and < 10 in one study applying MADRS [24].

Methodological quality of the included studies was graded using the Scottish Intercollegiate Guidelines Network (SIGN) checklist for controlled trials [25]. The instrument is composed of 13 questions, which are grouped into the following sections: internal validity and overall assessment of the study. Studies are considered to present high quality when most or all the criteria are met, acceptable quality if more than half of the criteria are met, low quality if fewer than half of the criteria are met and unacceptable if the paper is not relevant to key question. The quality assessment was performed by a reviewer under the supervision of a senior professional, and discrepancies were discussed until an agreement was reached.

### Statistical analysis

We performed the statistical analysis in RStudio Version 2022.02.0. The proportion of patients in the response and the remission groups were converted to odds ratios (ORs) and risk ratios (RRs) with 95% confidence intervals (CIs), where values beyond 1 reflect greater improvement for patients in the treatment group. For reporting the results, we have selected RR over OR due to its enhanced informativeness considering the study design and its greater convenience for interpretation in the context of health-economic studies [26]. We assessed data heterogeneity using Cochran Q statistic and we measured the proportion of variance accountable to data heterogeneity using I^2^ statistic. Data were synthesised using random-effects model, which accounts for possible heterogeneity of the data. The year of publication, number of sessions, number of pulses, and the interaction of number of pulses and sessions were included as moderator effects in the models to test for the effects of study characteristics on outcomes. Effect sizes and weights were visualised in forest plots. Publication bias was assessed with Begg and Mazumdar’s rank correlation test and visualised using funnel plots, in which effect size is plotted against standard errors estimated from study size [27].

## Results

### Study characteristics

**Fig. 1:**
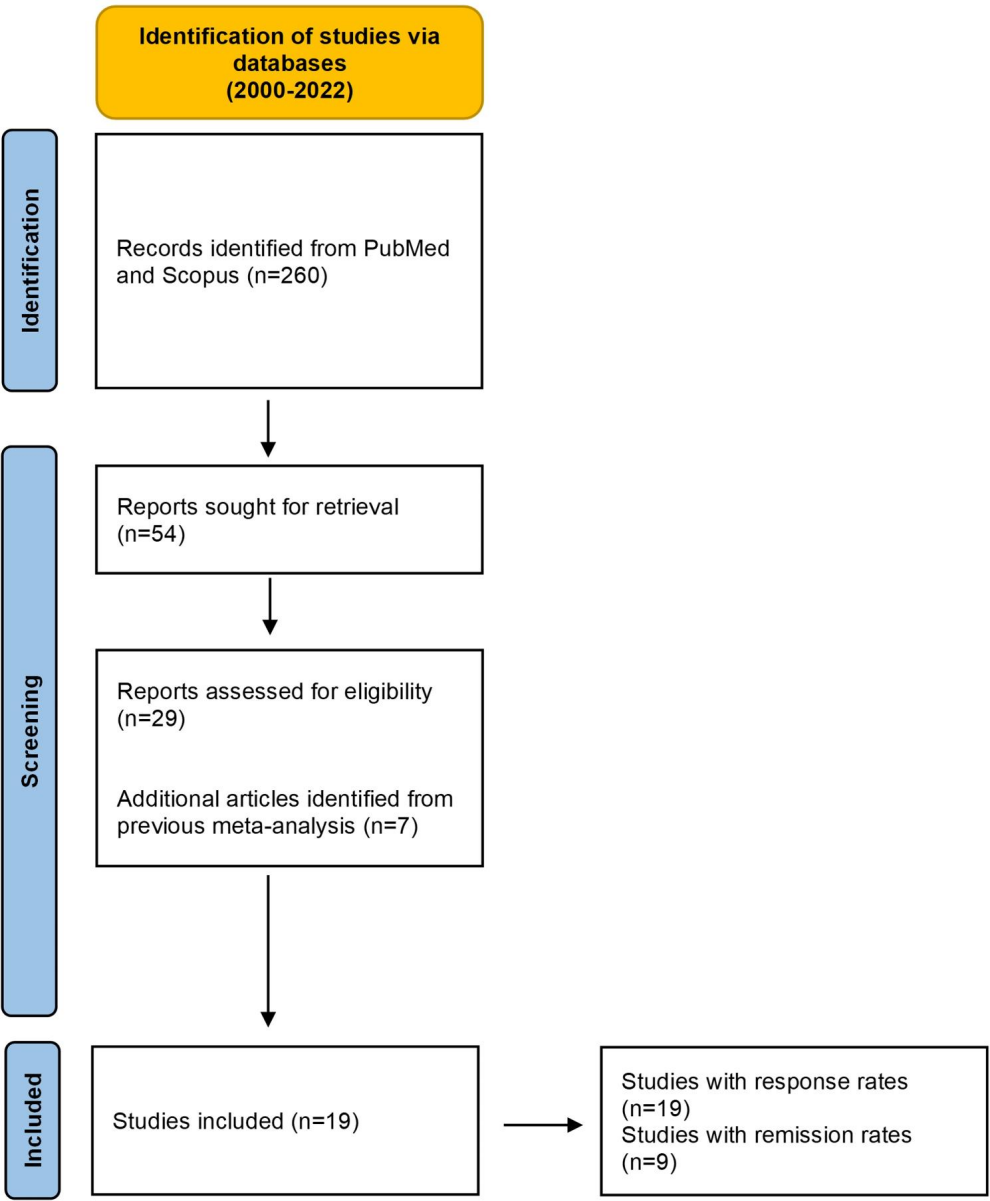
Flow diagram of study selection procedure

After deduplication of the hits in the two databases, a total of 260 publications were found. Subsequently, filtering the publications by title and abstract, 54 articles were obtained, while at the end of the full-text filtering process, 19 articles met the eligibility criteria (Fig. 1). A total of 19 RCTs on rTMS treatment for TRD were included in the meta-analysis. All of them reported response data [16–24, 28–37] and among them 9 reported remission rates as well [16–24], accounting for 854 and 551 subjects, respectively. Altogether, 840 participants were in the active rTMS group (mean age = 47.77 S.D.=6.9 years, 52.5% females) and 565 in the sham rTMS group (mean age = 47.79 S.D.=6.8 years, 50.3% females) in both response and remission articles (Table 1). Further characteristics of RCTs included in the study are listed in Appendix 1.

**Table 1:** The number of publications (n = 19) and subjects (n = 1405) included in the meta-analysis in terms of response or remission rates reported and active or sham rTMS applied

	Response rates reported	Response and Remission rates reported	Total number	Active rTMS	Sham rTMS
Number of publications	19	9	19	19	19
Number of Subjects	854	551	1405	840	565

The stimulation frequency in these studies ranged from 5 to 20 Hz and the intensity of stimulation was between 80% and 120% of the patients’ motor threshold (MT). Three studies used intensities less than 100% of motor threshold [29, 31, 36]. The total number of pulses during rTMS treatment ranged from 6000 to 120 000. Three studies [17, 30, 34] investigated two types of intensity (80% and 110%, 80% and 100%, 90% and 100% motor threshold) and did not report the combined data, therefore we included the higher intensity for continuous outcomes to avoid duplication in data analysis.

Assessment of the studies’ methodological quality shows that the included studies met at least half of the criteria presented in the SIGN checklist and, therefore, are considered acceptable or high-quality studies (2 high quality (++) and 17 acceptable (+) studies in a 4-level scale (High quality (++), Acceptable (+), Low quality (-), Unacceptable (0)) [25].

**Table 2:** Response and remission rates in those studies, which reported both response and remission rates (n = 9)

rTMS	No response (n = 368)	Response (n = 183)		Total (n = 551)
		Response without remission (n = 45)	Response with remission (n = 138)	
Active rTMS	187 (55.7%)	29 (8.6%)	120 (35.71%)	336 (100%)
Sham rTMS	181 (84.2%)	16 (7.44%)	18 (8.37%)	215 (100%)
RR		1.16 (95% CI 0.646–2.084)	4.27 (95% CI 2.68–6.79)	
NNT		84.09 (95 CI 17.22–28.59)	3.66 (95% CI 2.97–4.76)	

Risk Ratio (RR) and Numbers Needed to Treat (NNT) were calculated to show the difference between the active and the sham rTMS group

### Meta-analysis results: response rates

**Fig. 2:**
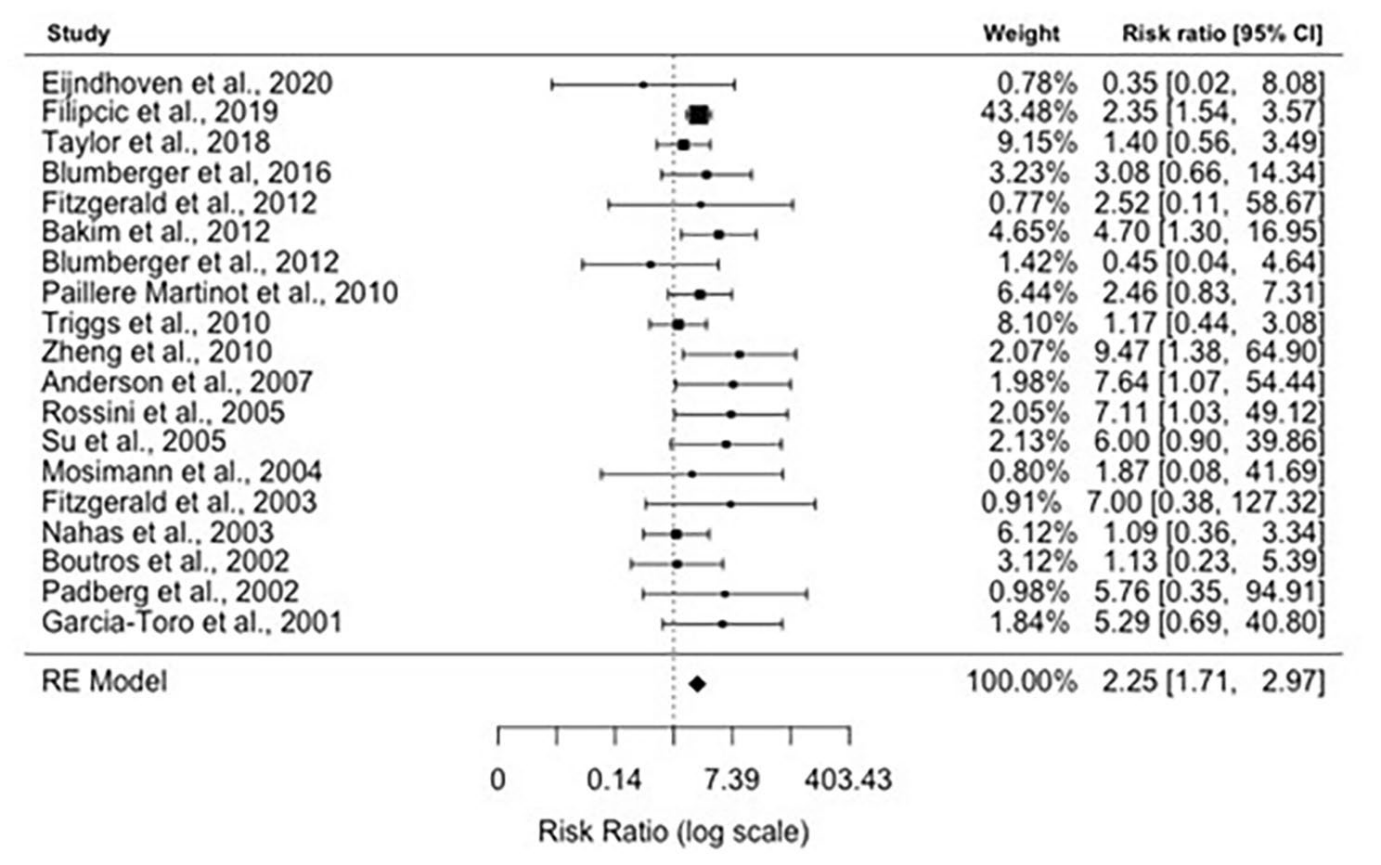
Forest plot depicting results of random-effects meta-analysis of studies reported response rates

**Fig. 3:**
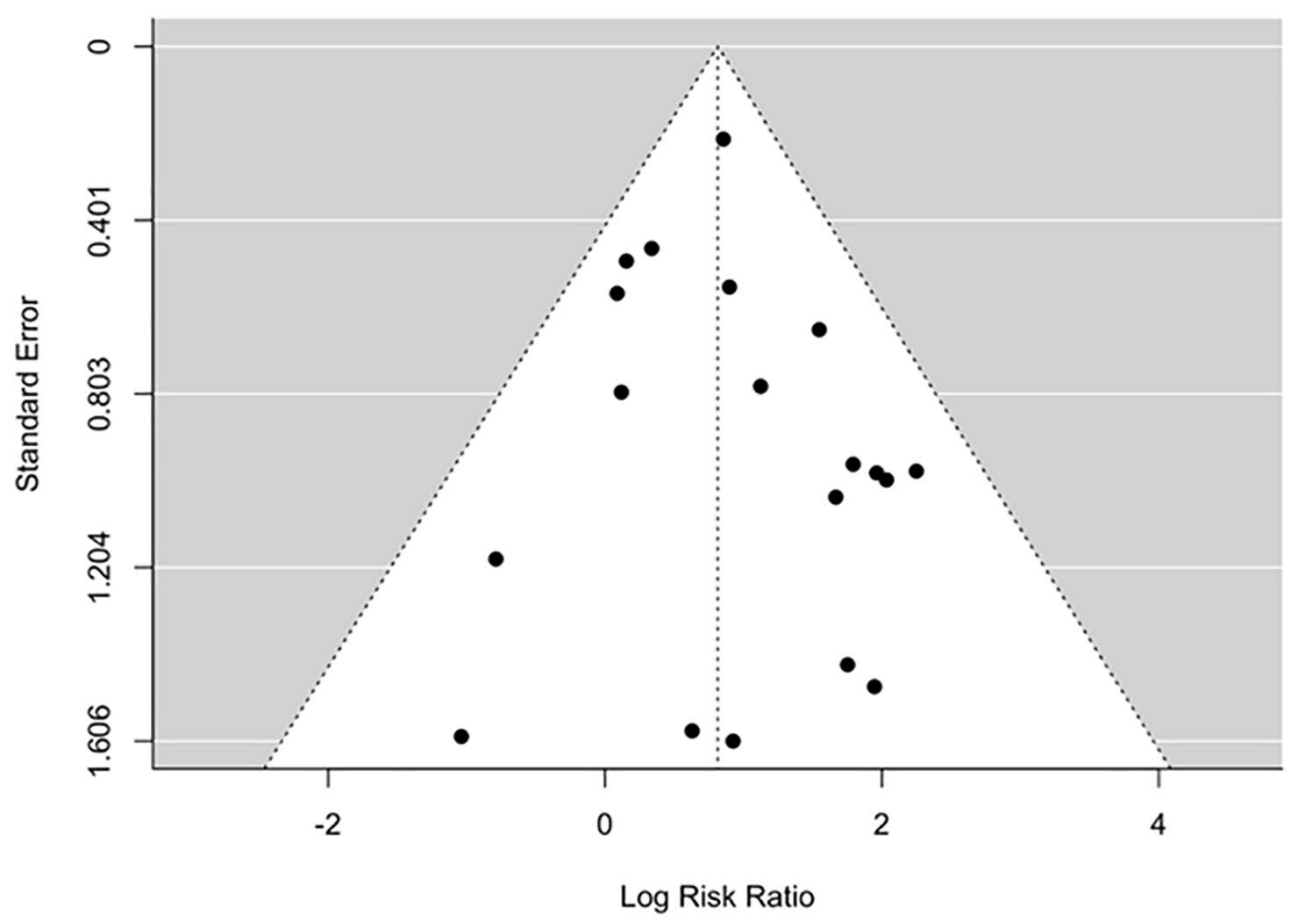
Funnel plot of studies reported response rates

Data on response rates were available from 19 studies. The overall response rate was 39.68% (200/504) in the active rTMS group and 13.71% (48/350) in the sham rTMS group. Figure 2. shows the forest plot of individual risk ratios (RR) with 95% CIs and weights of the 19 studies. The Random-Effects Model with Restricted Maximum Likelihood (REML) estimation (see comparison of goodness-of-fit values for all estimation methods in Appendix 2) shows significant positive association between rTMS treatment and response rates, treated patients being 2.25 times more likely to be in the response group compared to sham treatment (*RR: 2.25, 95% CI: 1.71–2.97)*. We identified no significant heterogeneity between studies *(I*^*2*^ *= 0.00%, Q = 17.53, df = 18, P = 0.49)*. The symmetrical distribution of the studies on the funnel plot (Fig. 3) and the non-significant result of the Begg’s adjusted rank correlation test *(Kendall’s tau = 0.06, p = 0.73)* suggest no publication bias. The inclusion of moderators including the year of publication, number of sessions, and the number of pulses in another mixed-effects model did not yield significant results (see model output in Appendix 3).

### Meta-analysis results: remission rates

**Fig. 4:**
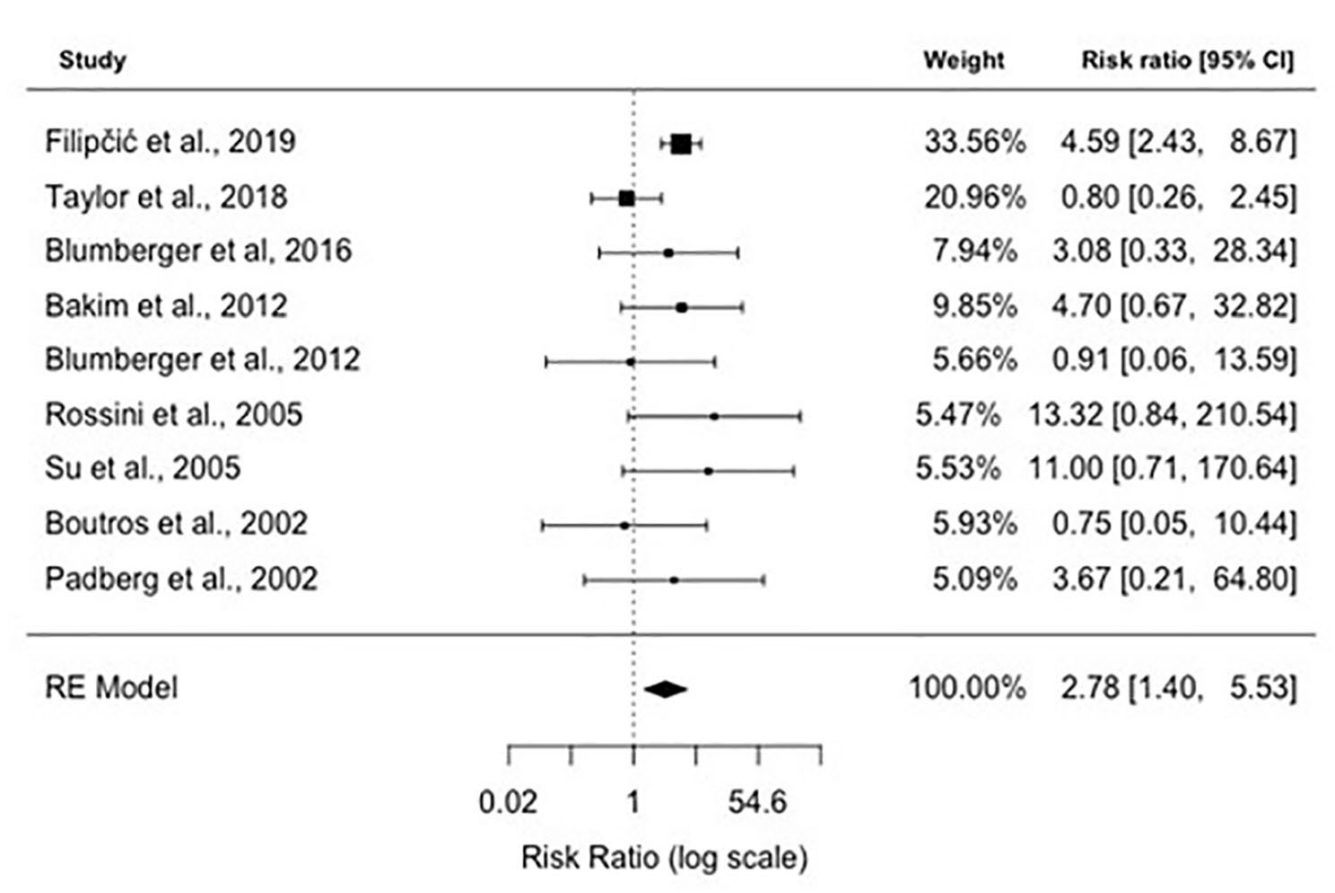
Forest plot depicting results of random-effects meta-analysis of studies reported remission rates

**Fig. 5:**
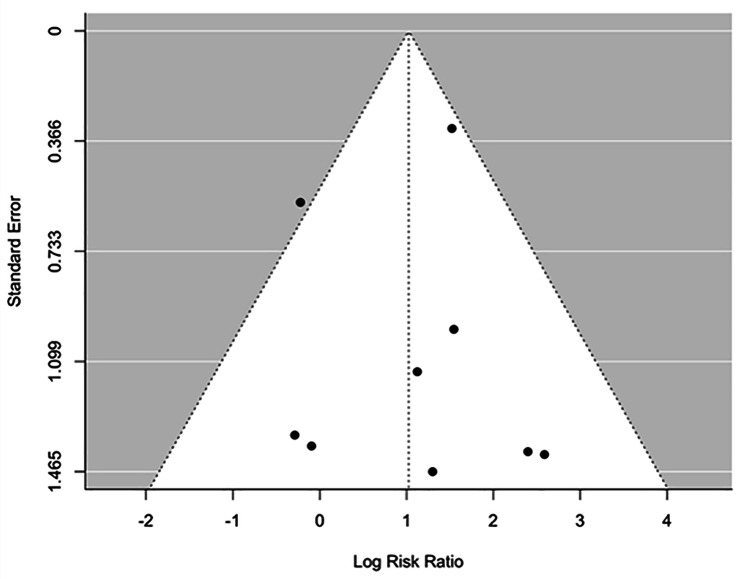
Funnel plot of studies reported remission rates

Data on remission rates were available from 9 studies. The overall remission rate was 35.71% (120/336) in the active rTMS group and 8.37% (18/215) in the sham rTMS group (Table 2). Figure 4. shows the forest plot of individual RRs with 95% CIs and weights of the 9 studies. The Random-Effects Model with Restricted Maximum Likelihood (REML) estimation (see comparison of goodness-of-fit values for all estimation methods in Appendix 4) shows significant positive association between rTMS treatment and remission rates, treated patients being 2.78 times more likely to be in the remission group compared to the sham treated control group *(RR: 2.78, 95% CI: 1.40–5.53)*. We identified no significant heterogeneity between studies *(I*^*2*^ *= 26.10%, Q = 11.10, df = 9, P = 0.20)*. The symmetrical distribution of the studies on the funnel plot (Fig. 5) and the non-significant result of the Begg’s adjusted rank correlation test *(Kendall’s tau = 0.11, p = 0.76)* suggest no publication bias. The inclusion of moderators including the year of publication, number of sessions, and the number of pulses in another mixed-effects model did not yield significant results (see model output in Appendix 5).

## Discussion

The results of the presented meta-analysis of 19 studies revealed that nearly 40% of TRD patients responded to the adjunctive active rTMS treatment, while with sham-rTMS (placebo group) this rate was just over 10%, meaning that the response rate was almost two and a half times more with active rTMS treatment (RR: 2.25, NNT: 6.1). This difference was even more pronounced when analyzing remission rates. Based on the 9 analyzed studies, nearly 36% of the TRD patients with rTMS treatment achieved remission, while barely 8% in the placebo group (sham-rTMS). Those with active rTMS treatment were almost three times more likely to achieve complete remission than those who did not receive active rTMS treatment (RR: 2.78, NNT: 5.4). An even greater difference was found when assessing the latter 9 studies for rTMS responders and remitters with RR of 1.16 and 4.27, respectively (Table 2.). These ratios show similarities with the results of previous meta-analyses however, the effect size is slightly smaller than in the former published literature [9]. Nevertheless, the value of these presented results is underlined by the fact that the strict eligibility criteria and the sophisticated statistical methods have helped to overcome the major methodological issues (e.g., heterogeneous patient population, differences in rTMS protocols and methods used to evaluate improvement) that limited the validity and generalizability of the conclusions from previous analyses [9]. The potential contributing factors in the background of the differences in the effect sizes in the current and the previous analyses are discussed below.

Previous meta-analyses had some limitations we aimed to eliminate in this study. Major limitations are associated with the *heterogeneity of the study populations and the rTMS protocols* applied across different studies. Although in 2008 FDA approved rTMS monotherapy in MDD [38], recent clinical practice applies rTMS generally as an add-on treatment to antidepressants. In this analysis, only those studies were included in which rTMS was used as an adjunctive treatment to antidepressants. Furthermore, in the past years, therapeutic guidelines included rTMS as a treatment alternative in patients with MDD, who did not respond well to antidepressant medication(s) [39]. Therefore, we investigated the impact of rTMS treatment only in patients with TRD, defined as an inadequate response to two previous antidepressant medications. Applying these inclusion criteria above provided a more homogeneous patient population and reflects everyday clinical practice.

Other major limitation of previous studies is the *heterogeneity of rTMS protocols* [40]. In this meta-analysis, we focused on the unilateral, high-frequency repetitive TMS technique, and included studies only with predefined specific rTMS parameters (such as 5–20 Hz, 10–30 sessions, 6000-120000 pulses, and 80–120% MT). However, similarly to Leggett et al. (2015) our deeper analysis revealed that there are no statistically significant differences between the various modalities of the rTMS technology, and the differences in the settings did not significantly affect the RR related to the efficacy [41].

The interpretation of several clinical trials using rTMS is also *limited by their sample size and composition* [40]. Furthermore, there is usually missing or incomplete information regarding the characteristics of subjects and their depression. Bipolar depression or mixed features in depression can be a confounding element as it can be an important reason for treatment resistance, and these patients may need more time to achieve remission [42, 43]. Hypothetically, subgroup analysis for bipolar depression may be a solution for this issue, however, with the currently available studies, this may lead to sample sizes unsuitable for meta-analysis. Therefore, Fornaro et al. (2020) recommended to set a proportion of patients with TRD and/or bipolar disorder as threshold for inclusion in future clinical trials [43]. This may also apply for the elderly population, however different rTMS protocols may be used for this population [44, 45].

Sham methods and blinding techniques in clinical trials with rTMS are other potential sources of bias as the different coil settings in degree, distance and rotation may influence the magnitude of placebo effect, generally resulting in smaller effect size difference between the active and sham group [46]. This, and the evolution of the blinding techniques can be an origin for the smaller effect size found in our study. The inclusion of sham coil (identical to the active coil), or configurations that could control both active and sham devices are generally common nowadays [24] while more than 10 years ago some studies included rTMS treatment naïve subjects, as control group [36]. Furthermore, in many studies the information regarding the blinding techniques is lacking or incomplete, stating that the evaluations were carried out by blinded raters [16–24, 28–33, 36, 37], or only mentioning double-blind study design [34, 35].

The primary aim of this meta-analysis was to assess the relative efficacy of rTMS treatment in TRD. Another purpose of conducting the meta-analysis was to use its results in analyzing the cost-effectiveness of rTMS compared with standard care as part of a health technology assessment (HTA) process to support decision-making on whether to include rTMS technology in public reimbursement. Therefore, the potential risks of publication bias were minimized with stringent inclusion criteria and with the application of various validated statistical methods to analyze heterogeneity and publication bias. The lack of significant heterogeneity and low publication bias makes our results reliable and may be used in cost-effectiveness analyses (CEAs) [47].

However, in contrast to a health technology assessment published in 2021 [48] our results showed greater risk ratio (efficacy) in remission than in response. The possible reason behind this different finding may be due to the more homogenous patient population included in this meta-analysis, as it may take longer for patients with bipolar disorder to reach complete remission compared to patients with MDD. Furthermore, our results may implicate a similar tendency as in the literature [49], that *patients who are not responding initially to rTMS may not reach remission*, however others with good initial response are more likely to achieve complete remission. This means, that early response to rTMS treatment may predict full remission with full-length (20–30 sessions) treatment. According to this, the assessment and the evaluation of the initial response to rTMS treatment may be included in the rTMS protocols to find the patient population who will most likely benefit from the rTMS treatment.

These results also implicate, that there is a subgroup of patients, who responded well to rTMS adjunctive treatment, meaning that they are not only responding to the treatment with add-on rTMS, but *they may reach complete remission*. From a clinical perspective, it may indicate that this sub-group of patients with partial response to antidepressants can achieve complete remission with adding rTMS. With other words, ‘rTMS may push these antidepressant partial responders to complete remission’.

It is interesting to interpret our results in the light of a recent meta-analysis of *antidepressant treatment in terms of efficacy compared to placebo*, published by Cipriani et al. in 2018. Based on 522 trials, the OR ranged between 1.15 and 1.55 for different antidepressants against placebo [50]. The relative efficacy of rTMS in this patient group is comparable or even better than antidepressant therapy. That is important, because patients with TRD are less likely to respond to further antidepressant therapy, whether it belongs to another pharmacological class or not. For these patients, neuromodulation technologies, like rTMS may be a rational treatment option as an add-on therapy or even in monotherapy [51].

The main limitations of our findings are originated from the aforementioned weaknesses of the RCTs on rTMS treatment and the controversies regarding the definition of TRD. The latter was based on the number of unsuccessful antidepressant therapies, as defined by the EMA. The blinding technique during the sham rTMS was not incorporated in our analysis, as most of the clinical studies did not provide detailed information or description on it. The inclusion of different studies with a slightly varying cut-off points and duration of remission may contribute to the small difference between remission and response rates, and it should be also highlighted as a limitation. Further shortcoming is that several former studies (especially before the year of 2010) did not separate between patients with bipolar and unipolar depressive disorders and the ratio of patients with bipolar depression in the study population varied from 8 to 50% [16, 22, 30, 31, 35, 36], which may result in smaller effect of the rTMS treatment. In this analysis, studies included more than 20% patients with bipolar disorder were excluded.

The results of this meta-analysis based on recent RCTs proved that rTMS treatment may be used effectively in the treatment of TRD, as it offers the possibility of achieving not only a symptomatic response, but also a complete remission in patients, who respond well to the treatment. In order to obtain reliable information regarding the effective applicability of rTMS in different subgroups of patients with MDD and to identify the patient population that would benefit most from rTMS treatment, further research is needed in this field with more precise methodology, including standardized treatment protocol and multi-centre study design, with further subgroup analyses in larger numbers of subjects and with possible predictor assessments (e.g. biomarkers, baseline cognitive performance) [52, 53]. Furthermore, recent studies and consequently the meta-analyses include treatment response and remission as primary outcome measures, however functioning and quality of life (QoL) would be also suitable measures to assess the efficacy and also the effectiveness of rTMS [40].

## Conclusions

Several meta-analyses proved the efficacy of rTMS treatment in MDD, which is comparable to pharmacotherapy, and may have even better tolerability. Our results strengthened that rTMS is associated with clinically relevant antidepressant effect in TRD as well, and may be a beneficial tool in the add-on treatment of patients with TRD. Furthermore, rTMS adjunctive treatment to antidepressants was found to be specifically effective in achieving full remission. The additional optimization of outcome parameters, novel stimulation and blinding techniques with the application of standardized protocols is a prerequisite for reliable evidence. Further research is needed to identify the patient population that will benefit most from the rTMS treatment. In addition, high quality meta-analyses can also support the conduct of health technology assessments, which can help to secure the reimbursement needed for their application in everyday clinical practice.

## Electronic supplementary material

Below is the link to the electronic supplementary material.

**Supplementary Material 1**: **Appendix 1**. Number of patients and patient characteristics in the different groups (response or remission and active or sham rTMS) by studies. **Appendix 2**. Comparison of goodness-of-fit values for all estimation methods in random-effects model estimating response rates. **Appendix 3**. Mixed-effects model with moderators estimating response rates. **Appendix 4**. Comparison of goodness-of-fit values for all estimation methods in random-effects model estimating remission rates. **Appendix 5**. Mixed-effects model with moderators estimating remission rates

## Data Availability

All data generated or analysed during this study are included in this published article [and its supplementary information files]. Competing interests.

## Abbreviations

CEA: Cost-Effectiveness Analysis
CI: Confidence Interval
EMA: European Medicines Agency
HAM-D/HDRS: Hamilton Depression Rating Scale
HCRU: Health-Care Resource Use
HFL: High-Frequency Left-sided
HTA: Health Technology Assessment
MADRS: Montgomery-Asberg Depression Rating Scale
MDD: Major Depressive Disorder
MT: Motor Threshold
NNT: Number Needed to Treat
OR: Odds Ratio
QoL: Quality of Life
RCT: Randomized Clinical Trial
REML: Random-Effects Model with Restricted Maximum Likelihood
RR: Risk Ratio
rTMS: repetitive Transcranial Magnetic Stimulation
SD: Standard Deviation
SIGN: Scottish Intercollegiate Guidelines Network
SLR: Systematic Literature Review
TRD: Treatment-Resistant Depression
