# Supplementary material for: Efficacy of repetitive transcranial magnetic stimulation (rTMS) adjunctive therapy for major depressive disorder (MDD) after two antidepressant treatment failures: meta-analysis of randomized sham-controlled trials
Source: BMC Psychiatry. 2023 Jul 27;23:545. doi: 10.1186/s12888-023-05033-y (PMC10375664; doi:10.1186/s12888-023-05033-y)
Supplement: Supplementary file 1 — Supplementary Material 1: Appendix 1. Number of patients and patient characteristics in the different groups (response or remission and active or sham rTMS) by studies. Appendix 2. Comparison of goodness-of-fit values for all estimation methods in random-effects model estimating response rates. Appendix 3. Mixed-effects model with moderators estimating response rates. Appendix 4. Comparison of goodness-of-fit values for all estimation methods in random-effects model estimating remission rates. Appendix 5. Mixed-effects model with moderators estimating remission rates [file 12888_2023_5033_MOESM1_ESM.docx]

Supplementary Material

Appendix 1: Number of patients and patient characteristics in the different groups (response or remission and active or sham rTMS) by studies

|  |  |  |  |  | **Response** | | | | **Remission** | | | |
| --- | --- | --- | --- | --- | --- | --- | --- | --- | --- | --- | --- | --- |
|  |  |  |  |  | **Active rTMS arm** | | **Sham rTMS arm** | | **Active rTMS arm** | | **Sham rTMS arm** | |
| **Study** | **Age, years (S.D. or median) in active rTMS arm** | **Age, years (S.D. or median) in sham rTMS arm** | **Female/male, n in active rTMS arm** | **Female/male, n in sham rTMS arm** | **event N°** | **total N°** | **event N°** | **total N°** | **event N°** | **total N°** | **event N°** | **total N°** |
| Eijndhoven, et al. , 2020 | 47.3 (11.5) | 49.7 (11.0) | 7/8 | 13/3 | 0 | 15 | 1 | 6 | NR | NR | NR | NR |
| Filipčić et al., 2019 | 50.5 (42-60) | 53 (48-61) | 75/72 | 45/36 | 81 | 147 | 19 | 81 | 75 | 147 | 9 | 81 |
| Taylor et al., 2018 | 46.9 (10.7) | 44.13 (11.1) | 11/5 | 10/6 | 7 | 16 | 5 | 16 | 4 | 16 | 5 | 16 |
| Blumberger et al, 2016 | 46.5 (14.1) | 48.1 (12) | 30/10 | 24/17 | 6 | 40 | 2 | 41 | 3 | 40 | 1 | 41 |
| Fitzgerald et al., 2012 | 43.4 (12.7) | 44.9 (15.7) | 15/9 | 8/12 | 1 | 24 | 0 | 20 | NR | NR | NR | NR |
| Bakim et al., 2012 | 40.9 (9.1) | 44.4 (10.2) | 20/3 | 11/1 | 18 | 23 | 2 | 12 | 9 | 23 | 1 | 12 |
| Blumberger et al., 2012 | 48.9 (13.4) | 45.8 (13.4) | 12/10 | 14/6 | 1 | 22 | 2 | 20 | 1 | 22 | 1 | 20 |
| Paillere Martinot et al., 2010 | 48.2 (7.8) | 46.6 (10.3) | 11/7 | 10/4 | 10 | 18 | 3 | 14 | NR | NR | NR | NR |
| Triggs et al., 2010 | 46.7 (15.3) | 41.9 (14.1) | 14/4 | 2/5 | 9 | 18 | 3 | 7 | NR | NR | NR | NR |
| Zheng et al., 2010 | 26.9 (6.2) | 26.7 (4.3) | 7/12 | 5/10 | 12 | 19 | 1 | 15 | NR | NR | NR | NR |
| Anderson et al., 2007 | 48 (8) | 46 (12) | 7/4 | 9/5 | 6 | 11 | 1 | 14 | NR | NR | NR | NR |
| Rossini et al., 2005 | 55.7 (9.9.) | 56.3 (12.6) | 27/10 | 11/6 | 16 | 36 | 1 | 16 | 14 | 36 | 0 | 16 |
| Su et al., 2005 | 43.4 (11.3) | 42.6 (11) | 15/5 | 7/3 | 12 | 20 | 1 | 10 | 10 | 20 | 0 | 10 |
| Mosimann et al., 2004 | 60 (13.4) | 64.4 (13) | 5/10 | 5/4 | 1 | 15 | 0 | 9 | NR | NR | NR | NR |
| Fitzgerald et al., 2003 | 42.2 (9.8) | 49.1 (14.2) | 8/12 | 11/9 | 3 | 20 | 0 | 20 | NR | NR | NR | NR |
| Nahas et al., 2003 | 42.4 (7.3) | 43.4 (9.3) | 7/4 | 7/5 | 4 | 11 | 4 | 12 | NR | NR | NR | NR |
| Boutros et al., 2002 | 49.4 (8) | 52 (7) | 4/8 | 1/8 | 3 | 12 | 2 | 9 | 1 | 12 | 1 | 9 |
| Padberg et al., 2002 | 61.2 (4.6) | 52.7 (5.7) | 7/13 | 8/2 | 5 | 20 | 0 | 10 | 3 | 20 | 0 | 10 |
| Garcia-Toro et al., 2001 | 50 (11) | 51.5 (15.9) | 8/10 | 7/10 | 5 | 17 | 1 | 18 | NR | NR | NR | NR |

Appendix 2: Comparison of goodness-of-fit values for all estimation methods in random-effects model estimating response rates

|  | **DL** | **HE** | **HS** | **HSk** | **SJ** | **ML** | **REML** | **EB** | **PM** | **GENQ** |
| --- | --- | --- | --- | --- | --- | --- | --- | --- | --- | --- |
| logLik: | -23.48 | -23.48 | -23.48 | -23.48 | -25.38 | -23.48 | -23.48 | -23.48 | -23.48 | -23.81 |
| deviance: | 17.53 | 17.53 | 17.53 | 17.53 | 21.33 | 17.53 | 17.53 | 17.53 | 17.53 | 18.19 |
| AIC: | 50.96 | 50.96 | 50.96 | 50.96 | 54.76 | 50.96 | 50.96 | 50.96 | 50.96 | 51.62 |
| BIC: | 52.85 | 52.85 | 52.85 | 52.85 | 56.65 | 52.85 | 52.85 | 52.85 | 52.85 | 53.51 |
| AICc: | 51.71 | 51.71 | 51.71 | 51.71 | 55.51 | 51.71 | 51.71 | 51.71 | 51.71 | 52.37 |

Appendix 3: Mixed-effects model with moderators estimating response rates

Mixed-Effects Model (k = 19; tau^2 estimator: REML)

logLik deviance AIC BIC AICc

-19.2628 38.5257 50.5257 54.3600 62.5257

tau^2 (estimated amount of residual heterogeneity): 0.0486 (SE = 0.1912)

tau (square root of estimated tau^2 value): 0.2204

I^2 (residual heterogeneity / unaccounted variability): 7.56%

H^2 (unaccounted variability / sampling variability): 1.08

R^2 (amount of heterogeneity accounted for): 0.00%

Test for Residual Heterogeneity:

QE(df = 14) = 14.5887, p-val = 0.4068

Test of Moderators (coefficients 2:5):

QM (df = 4) = 2.7186, p-val = 0.6060

Model Results:

estimate se zval pval ci.lb ci.ub

intrcpt 67.7607 143.3071 0.4728 0.6363 -213.1160 348.6374

year -0.0330 0.0721 -0.4570 0.6477 -0.1744 0.1084

sessions -0.0252 0.1415 -0.1782 0.8585 -0.3025 0.2521

pulses -0.0001 0.0001 -0.5973 0.5503 -0.0003 0.0002

sessions:pulses 0.0000 0.0000 0.6132 0.5398 -0.0000 0.0000

---

Signif. codes: 0 ‘***’ 0.001 ‘**’ 0.01 ‘*’ 0.05 ‘.’ 0.1 ‘ ’ 1

Appendix 4: Comparison of goodness-of-fit values for all estimation methods in random-effects model estimating remission rates

|  | **DL** | **HE** | **HS** | **HSk** | **SJ** | **ML** | **REML** | **EB** | **PM** | **GENQ** |
| --- | --- | --- | --- | --- | --- | --- | --- | --- | --- | --- |
| logLik: | -13.30 | -13.93 | -13.40 | -13.31 | -13.45 | -13.29 | -13.33 | -13.35 | -13.35 | -13.96 |
| deviance: | 9.84 | 11.10 | 10.04 | 9.86 | 10.14 | 9.83 | 9.91 | 9.94 | 9.94 | 11.17 |
| AIC: | 30.59 | 31.85 | 30.79 | 30.61 | 30.89 | 30.59 | 30.66 | 30.70 | 30.70 | 31.93 |
| BIC: | 30.99 | 32.25 | 31.19 | 31.01 | 31.29 | 30.98 | 31.06 | 31.09 | 31.09 | 32.32 |
| AICc: | 32.59 | 33.85 | 32.79 | 32.61 | 32.89 | 32.59 | 32.66 | 32.70 | 32.70 | 33.93 |

Appendix 5: Mixed-effects model with moderators estimating remission rates

Mixed-Effects Model (k = 9; tau^2 estimator: REML)

logLik deviance AIC BIC AICc

-8.0720 16.1439 28.1439 24.4617 112.1439

tau^2 (estimated amount of residual heterogeneity): 1.8393 (SE = 2.2629)

tau (square root of estimated tau^2 value): 1.3562

I^2 (residual heterogeneity / unaccounted variability): 64.04%

H^2 (unaccounted variability / sampling variability): 2.78

R^2 (amount of heterogeneity accounted for): 0.00%

Test for Residual Heterogeneity:

QE(df = 4) = 11.6967, p-val = 0.0198

Test of Moderators (coefficients 2:5):

QM(df = 4) = 0.3307, p-val = 0.9877

Model Results:

estimate se zval pval ci.lb ci.ub

intrcpt -145.6469 626.2264 -0.2326 0.8161 -1373.0280 1081.7343

year 0.0746 0.3148 0.2369 0.8127 -0.5425 0.6916

sessions -0.1266 0.4096 -0.3091 0.7572 -0.9295 0.6762

pulses -0.0002 0.0004 -0.4064 0.6844 -0.0009 0.0006

sessions:pulses 0.0000 0.0000 0.3731 0.7091 -0.0000 0.0000

---

Signif. codes: 0 ‘***’ 0.001 ‘**’ 0.01 ‘*’ 0.05 ‘.’ 0.1 ‘ ’ 1
